# Transcriptional regulation of matrix metalloproteinase-1 and collagen 1A2 explains the anti-fibrotic effect exerted by proteasome inhibition in human dermal fibroblasts

**DOI:** 10.1186/ar2991

**Published:** 2010-04-29

**Authors:** Laurence Goffin, Queralt Seguin-Estévez, Montserrat Alvarez, Walter Reith, Carlo Chizzolini

**Affiliations:** 1 Immunology and Allergy, Department of Internal Medicine, Geneva University Hospital and School of Medicine, rue Gabrielle Perret-Gentil 4, 1211 Geneva 14, Switzerland; 2 Department of Pathology and Immunology, Geneva University Hospital and School of Medicine, rue Michel Servet 1, 1211 Geneva 14, Switzerland

## Abstract

**Introduction:**

Extracellular matrix (ECM) turnover is controlled by the synthetic rate of matrix proteins, including type I collagen, and their enzymatic degradation by matrix metalloproteinases (MMPs). Fibrosis is characterized by an unbalanced accumulation of ECM leading to organ dysfunction as observed in systemic sclerosis. We previously reported that proteasome inhibition (PI) *in vitro *decreases type I collagen and enhances MMP-1 production by human fibroblasts, thus favoring an antifibrotic fibroblast phenotype. These effects were dominant over the pro-fibrotic phenotype induced by transforming growth factor (TGF)-β. Here we investigate the molecular events responsible for the anti-fibrotic phenotype induced in fibroblasts by the proteasome inhibitor bortezomib.

**Methods:**

The steady-state mRNA levels of *COL1A1*, *COL1A2*, *TIMP-1*, *MMP-1*, and *MMP-2 *were assessed by quantitative PCR in human dermal fibroblasts cultured in the presence of TGF-β, bortezomib, or both. Transient fibroblast transfection was performed with wild-type and mutated *COL1A1 *and *MMP-1 *promoters. Chromatin immunoprecipitation, electrophoretic mobility shift assay (EMSA), and DNA pull-down assays were used to assess the binding of c-Jun, SP1, AP2, and Smad2 transcription factors. Immunoblotting and immunofluorescent microscopy were performed for identifying phosphorylated transcription factors and their cellular localization.

**Results:**

Bortezomib decreased the steady-state mRNA levels of *COL1A1 *and *COL1A2*, and abrogated SP1 binding to the promoter of *COL1A2 *in both untreated and TGF-β-activated fibroblasts. Reduced COL1A2 expression was not due to altered TGF-β-induced Smad2 phosphorylation, nuclear translocation, or binding to the *COL1A2 *promoter. In contrast to collagen, bortezomib specifically increased the steady-state mRNA levels of MMP-1 and enhanced the binding of c-Jun to the promoter of MMP-1. Furthermore, disruption of the proximal AP-1-binding site in the promoter of MMP-1 severely impaired MMP-1 transcription in response to bortezomib.

**Conclusions:**

By altering the binding of at least two transcription factors, c-Jun and SP1, proteasome inhibition results in increased production of MMP-1 and decreased synthesis of type I collagen in human dermal fibroblasts. Thus, the antifibrotic phenotype observed in fibroblasts submitted to proteasome inhibition results from profound modifications in the binding of key transcription factors. This provides a novel rationale for assessing the potential of drugs targeting the proteasome for their anti-fibrotic properties.

## Introduction

The extracellular matrix (ECM) provides a controlled environment for cellular differentiation and tissue development, thereby participating in the maintenance of organ morphology and function. ECM integrity results from a continuous and tightly regulated deposition and degradation of its components. Type I collagen is among the most abundant ECM proteins and its excessive dermal deposition is one of the key features of systemic sclerosis (SSc) (scleroderma), a prototypic fibrotic condition [1-3]. Type I collagen forms a characteristic triple-helix structure composed of two alpha1 subunits and one alpha2 subunit, encoded by the collagen 1A1 (*COL1A1*) and *COL1A2 *genes, of which the coordinated transcription rates ensure a 2:1 ratio [4].

Among various soluble molecules inducing the production of type I collagen, the most extensively studied is transforming growth factor-beta (TGF-β) [5,6]. TGF-β-responsive elements have been mapped in the -378/-183 region of the mouse and human *COL1A2 *promoter [7,8]. TGF-β-mediated increase in the production of type I collagen results from increased binding of transcription factors to three GC-rich SP1 sites (in the -303/-271 region) and one activation protein-1 (AP-1) site (-265/-241) within the *COL1A2 *promoter [9]. SP1 binding is essential since blocking SP1 recruitment by point mutations in the DNA consensus sequence leads to inhibition of type I collagen synthesis, and overexpression of SP1 stimulates both basal and TGF-β-mediated *COL1A2 *transcription [8]. Furthermore, Smad2/3 signaling molecules induced by TGF-β [10] bind to the SP1 consensus sequence in the *COL1A2 *promoter region. Smad2/3 interacts also with the transcriptional co-activators p300/CREB-binding protein (CBP), which enhance both basal and TGF-β-induced *COL1A2 *promoter activity [11].

Matrix metalloproteinases (MMPs) play a major role in ECM degradation. They are regulated at the transcriptional level and undergo post-transcriptional maturation and their catalytic activity is inhibited by tissue inhibitors of MMP (TIMPs) [12,13]. MMP-1 or interstitial collagenase unwinds native type I collagen and initiate its degradation, whereas MMP-2 and MMP-9 are two gelatinases, which efficiently digest degraded collagen. Interestingly, these MMPs are not co-ordinately regulated. Tumor necrosis factor-alpha (TNF-α) enhances MMP-1 [14] and MMP-9 [15] expression, whereas TGF-β enhances MMP-2 [16] and MMP-9 [17] synthesis but decreases MMP-1 production [18].

The proteasome is a barrel-shaped, multi-catalytic protease complex present in the cytosol and in the nuclei and triggers degradation of multi-ubiquitinated proteins [19]. It maintains cell homeostasis by promoting clearance of damaged or improperly folded proteins and degrades key components involved in the cell cycle and cell signaling [20]. We recently reported that proteasome inhibition (PI) profoundly modifies the phenotype of human dermal fibroblasts by reducing type I collagen synthesis and increasing MMP-1 production [21]. This effect was dominant on the pro-fibrotic activity of TGF-β and observed in normal as well as SSc fibroblasts. Furthermore, PI induced the phosphorylation, accumulation, and nuclear translocation of c-Jun. These *in vitro *characteristics are consistent with the anti-fibrotic activity exerted by PI in many, but not all, *in vivo *models of fibrosis [22-27].

In the present study, we aimed at dissecting the molecular mechanisms involved in the anti-fibrotic activity of PI on human dermal fibroblasts. We inhibited the proteasome with bortezomib, a highly specific and potent PI used in humans as a therapeutic agent for multiple myeloma [28-30]. We provide evidence that the anti-fibrotic property of PI results from both the induction of *MMP-1 *expression via proximal AP-1 sites and the repression of *COL1A2 *transcription via SP1 sites.

## Materials and methods

### Cell culture

A primary human fibroblast cell line was established from skin punch biopsies of a healthy donor, as described previously [21]. Permission to perform this investigation was granted by the ethics committee of our institution. Informed consent was obtained in accordance with the Declaration of Helsinki. Fibroblasts were maintained in Dulbecco's modified Eagle's medium (Invitrogen Corporation, Carlsbad, CA, USA), supplemented with 10% fetal calf serum (FCS) (Sigma-Aldrich, St. Louis, MO, USA), 2 mM glutamine (Invitrogen Corporation), non-essential amino acids (Invitrogen Corporation), 50 U/mL penicillin, and 50 μg/mL streptomycin (Invitrogen Corporation) and grown in 5% CO_2 _at 37°C. Fibroblasts used at passages 5 to 14 were grown up to 80% confluence, starved overnight in medium containing 1% FCS, and then cultured in the presence of TGF-β (5 ng/mL), bortezomib (1 μM), or TNF-α (10 ng/mL) for the desired periods of time.

### Reagents and antibodies

Bortezomib (PS-341, Velcade) was from Millennium Pharmaceuticals (Cambridge, MA, USA). TGF-β and TNF-α were from R&D Systems, Inc. (Minneapolis, MN, USA). Immunolabeling and immunoprecipitation were performed using anti-type I collagen (SouthernBiotech, Birmingham, AL, USA), anti-MMP-1 (Chemicon International Inc., Temecula, CA, USA), anti-phospho-c-Jun, anti-phospho-Smad2, and anti-c-Jun (Cell Signaling Technology, Inc., Beverly, MA, USA), anti-AP2, anti-SP1, anti-p300, anti-Ets1, and anti-TFIIEα (Santa Cruz Biotechnology, Inc., Santa Cruz, CA, USA), anti-Smad2/3 (Upstate, now part of Millipore Corporation, Billerica, MA, USA), anti-β-tubulin (Sigma-Aldrich) primary antibodies, and anti-goat (The Binding Site, Birmingham, UK) or anti-rabbit or anti-mouse (DakoCytomation, Baar, Switzerland) IgG antibodies coupled to horseradish peroxidase. For DNA pull-down assays, streptavidin agarose beads were from Pierce Biotechnology (Rockford, IL, USA).

### Quantitative reverse transcription-polymerase chain reaction

Total RNA was extracted with TRIzol reagent (Invitrogen Corporation), and 1 μg was reverse-transcribed using random primers and Superscript II (Invitrogen Corporation). Real-time polymerase chain reaction (PCR) was performed with an ABI PRISM SDS 7900 instrument (Applied Biosystems, Foster City, CA, USA) using TaqMan probes (Applied Biosystems) and with an ABI PRISM SDS 7700 instrument using an SYBR-Green based kit for quantitative PCR (Eurogentec, Liege, Belgium). For each sample, gene expression was normalized using human elongation factor 1 mRNA (HsEEF1A1). Primers used for PCR are listed in Table 1.

**Table 1:** Oligonucleotide sequences

Name	Sequence
Electrophoretic mobility shift assay	
MMP-1 AP-1 S	CTAGTGA**TGAGTCA**GCCGGATC
MMP-1 AP-1 AS	GATCCGGC**TGACTCA**TCACTAG

Chromatin immunoprecipitation	
MMP-1 AP-1 fw	CCTCTTGCTGCTCCAATATC
MMP-1 AP-1 rv	TCTGCTAGGAGTCACCATTTC
MMP-2 AP2 fw	GTGGAGGAGGGCGAGTAGGG
MMP-2 AP2 rv	CTGGGAGGGAGCTGGCAGAG
MMP-9 AP-1 fw	GAGAGGAGGAGGTGGTGTAAG
MMP-9 AP-1 rv	TTAAGGAGGCGCTCCTGTG
COL1A1 -200/+100 fw	CAGAGCTGCGAAGAGGGGA
COL1A1 -200/+100 rv	AGACTCTTTGTGGCTGGGGAG
COL1A2 fw2	GCGGAGGTATGCAGACAACG
COL1A2 rv1	GGGCTGGCTTCTTAAATTG
MMP-1 ORF fw	TAAGTACTGGGCTGTTCAGG
MMP-1 ORF rv	GAGCAGCATCGATATGCTTC
COL1A2 ORF fw	GCCCTCAAGGTTTCCAAG
COL1A2 ORF rv	GGGAGACCCATCATTTCAC

Reverse transcription-polymerase chain reaction	
COL1A1	Hs00164004_m1
COL1A2	Hs00164099_m1
TIMP-1	Hs00171558_m1
MMP-1	Hs00233958_m1
MMP-2	Hs00234422_m1
HsEEF1A1	CACCTGAGCAGTGAAGCCAGCTGCTT

DNA pull-down assay	
Biotin-MMP-1-S	GATCGAGAGGATGTTATAAAGCATGAGTCAG
Biotin-MMP-1-AS	CTGACTCATGCTTTATAACATCCTCTCGATC
Biotin-COL1A2-S	GAAAGGGCGGGGGAGGGCGGGAGGATGCGGAGGGCGGAG
Biotin-COL1A2-AS	CTCCGCCCTCCGCATCCTCCCGCCCTCCCCCGCCCTTTC

Bold case indicates proximal activation protein-1 (AP-1) site. Underlining indicates transcription binding sites (Ets and AP-1 for matrix metalloproteinase-1 [MMP-1] and SP1 for collagen 1A2 [COL1A2]).

### Immunoblotting and enzyme-linked immunosorbent assay

Type I collagen, MMP-1, MMP-2, and TIMP-1 proteins were quantified in the supernatants of fibroblasts submitted to various culture conditions for 48 hours. Type I collagen and MMP-1 were quantified by immunoblotting in culture supernatants concentrated to one tenth of their original volume using Vivaspin 6-mL concentrators (Sartorius AG, Goettingen, Germany). Total protein (20 μg) was resolved by SDS-PAGE, transferred onto nitrocellulose membranes (Hybond; Amersham Biosciences, now part of GE Healthcare, Little Chalfont, UK), and immunoblotted with specific antibodies [21]. Signals were revealed according to enhanced chemiluninescence (ECL) protocols (GE Healthcare) and quantified by phosphor imaging. TIMP-1 and MMP-2 were quantified by enzyme-linked immunosorbent assay in accordance with the instructions of the manufacturer (R&D Systems, Inc.).

### Transient cell transfection and reporter gene assays

Plasmids carrying the Renilla luciferase gene under control of the constitutive Herpes simplex virus thymidine kinase (TK) promoter (pGL4.74) and plasmids carrying the firefly luciferase gene under control of the constitutive SV40 promoter (pLG4.13) were purchased from Promega Corporation (Madison, WI, USA). The pLG2-derived plasmids, containing the wild-type (TGACTCA) or variant (TTACGTCA) AP-1 site situated at -72 of the *MMP-1 *promoter, were kindly provided by Franck Verrecchia (Hôpital Saint-Louis, Paris, France) [31]. The pLG2-derived plasmids containing the wild-type (-311/+114) or deleted (-112/+114) *COL1A1 *promoter were kindly provided by Philippe Galera (CHU, Caen, France) [32]. A promoter-free plasmid encoding firefly luciferase (pLG4.10) was used as a negative control. The day before transfection, fibroblasts were seeded in six-well plates to reach 80% confluence. Various combinations of plasmid DNA (1 μg total) and 3 μL of transfection reagent (Transfast from Promega Corporation) were added to 1 mL of medium containing 1% FCS and mixed vigorously. The transfection mix contained Renilla luciferase and firefly luciferase plasmids at a ratio of 1 to 10. After 10 minutes at room temperature, the cultures were placed for 1 hour at 37°C, 2 mL of medium containing 10% FCS was added to each well, and the cells were incubated for 48 hours at 37°C. Cell lysis was performed as recommended by the manufacturer (Promega Corporation), and luciferase activities were measured with a Luminometer (Luminoskan Ascent^®^; Thermo LabSystems, now part of Thermo Electron Corporation, Waltham, MA, USA) using the Dual-Luciferase Reporter Assay from Promega Corporation. Firefly luciferase activity was normalized to that of Renilla luciferase. The corrected activities reflect the induction of the tested promoters.

### Chromatin immunoprecipitation

Chromatin was prepared and immunoprecipitated as previously described [33]. Immunoprecipitated DNA derived from 1 μg of input chromatin DNA and a series of standards containing 0.01 to 10 ng of total input chromatin DNA were analyzed by real-time PCR using primers listed in Table 1. The amount of immunoprecipitated DNA was calculated from a standard curve generated with the input chromatin. Real-time PCR amplifications were repeated in triplicate.

### Nuclear extracts

Fibroblasts were washed twice with ice-cold phosphate-buffered saline (PBS) and collected with a rubber policeman in 1 mL of PBS containing 1 mM ethylenediaminetetraacetic acid (EDTA). Cells were lysed for 15 minutes on ice in hypotonic buffer (10 mM Hepes pH 7.9; 10 mM KCl; 0.1 mM EDTA; 0.1 mM EGTA; 1 mM dithiothreitol [DTT]; 0.15 vol/vol complete protease inhibitor mix from Roche [Basel, Switzerland]; 0.2 mM phenylmethylsulfonyl fluoride [PMSF]; 100 nM okadaic acid; and 1 mM orthovanadate). The cell lysate was centrifuged at 13,000 rpm for 30 seconds, the supernatants were discarded, and the nuclei were suspended in extraction buffer (20 mM Hepes pH 7.9; 0.4 M NaCl; 1 mM EDTA; 1 mM EGTA; 1 mM DTT; 0.15 vol/vol complete protease inhibitor mix from Roche; and 0.2 mM PMSF). Samples were shaken vigorously for 15 minutes at 4°C and centrifuged for 5 minutes at 13,000 rpm, and the supernatants were collected.

### Electrophoretic mobility shift assay

Complementary oligonucleotides (listed in Table 1) were mixed in a 1:1 molar ratio at 100 pmole/μL, heated for 5 minutes at 95°C, and slowly cooled down to room temperature. Hybridized DNA probes were radiolabeled with γ-^32^P-ATP in the presence of T4 polynucleotide kinase (Invitrogen Corporation) and purified by chromatography using a Sephadex G-25 spin column (Roche). An aliquot of labeled probe (2 × 10^4 ^cpm) was incubated with 5 μg of nuclear extract in binding buffer (nuclear extraction buffer containing 130 ng/μL Poly dIdC, 0.7 mg/mL bovine serum albumin, and 15% glycerol) for 30 minutes at room temperature. Alternatively, the nuclear extract was pre-incubated for 30 minutes at room temperature prior to addition of the probe, with 1 μg of specific antibodies for supershift assays or with a 100-fold excess of cold probe for competition experiments. Protein-DNA complexes were resolved on non-denaturing 4% polyacrylamide gels, and radioactive bands were detected with x-ray films (Kodak BioMax MR film from Sigma-Aldrich).

### DNA pull-down assays

Complementary biotinylated oligonucleotides (listed in Table 1) were hybridized as described in the EMSA protocol. Annealed biotinylated DNA (1 μg) was incubated with 300 μg of nuclear extract in binding buffer (supplemented with 0.2 mg/mL sheared salmon DNA) for 30 minutes at room temperature, and streptavidin-agarose beads were then added in binding buffer (supplemented with 0.2 mg/mL sheared salmon DNA and 150 mM NaCl) for 3 hours at 4°C. Following three washes in blocking buffer, beads were boiled in Laemmli buffer and eluted proteins were resolved and immunoblotted as described above. Normalization to the total nuclear protein content was performed by Western immunoblotting of the input fractions with anti-TFIIEα antibody.

### Immunofluorescence

Fibroblasts were seeded on coverslips in six-well plates (5 × 10^4 ^cells per well). Cells were washed in PBS, fixed in 4% paraformaldehyde for 20 minutes at room temperature, incubated successively in 1 mM NH_4_Cl and 4% Tween, labeled with primary antibody for 1 hour, and then incubated with alexa488-coupled anti-rabbit IgG antibody (Invitrogen Corporation) for 45 minutes at room temperature. Subcellular localization was observed with a fluorescence microscope (Axiovert 200, Carl Zeiss, Gottingen, Germany), and photographs were taken at a magnification of × 40.

### Statistical analysis

The Student *t *test was used for statistical analysis. A *P *value of less than 0.05 was considered significant. To assess the normal distribution of our data, we assessed their skewness and kurtosis, which provided values consistent with normal distribution using the GraphPad Prism version 4.00 (GraphPad Software, Inc., San Diego, CA, USA) for Windows.

## Results

### Proteasome inhibition abrogates the production of type I collagen induced by TGF-β

**Figure 1:**
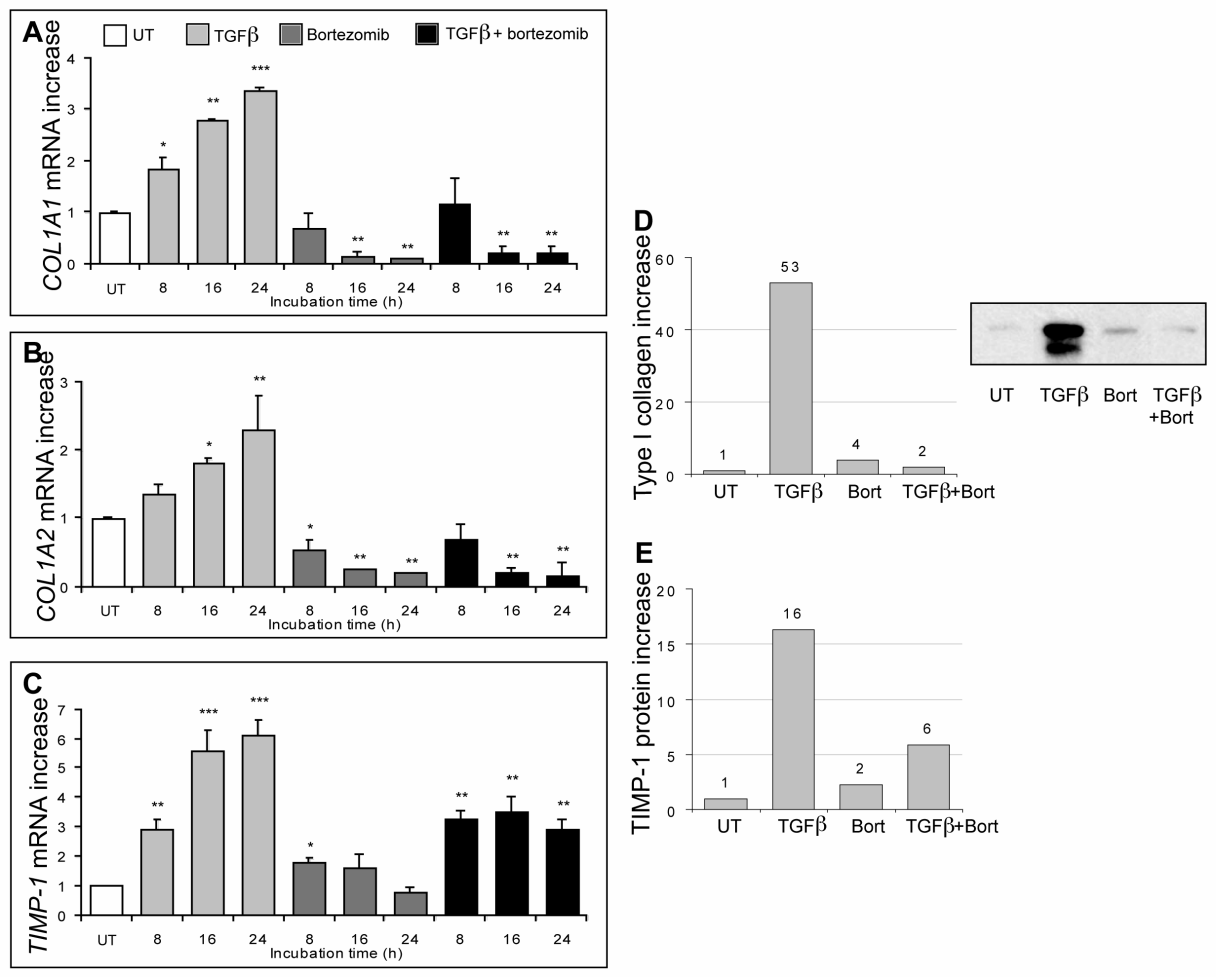
**Proteasome inhibition reverses the pro-fibrotic effects of TGF-β in dermal fibroblasts**. Fibroblasts were cultured in the presence of TGF-β (10 ng/mL) or bortezomib (1 μM) or both (TGF-β was added 1 hour after bortezomib) or were left untreated for the indicated amount of time **(a-c) **or for 48 hours **(d, e)**. mRNA levels for *COL1A1 *(a), *COL1A2 *(b), and *TIMP-1 *(c) were assessed by quantitative polymerase chain reaction and normalized to HsEEF1A1 mRNA levels. The increase in treated cells relative to untreated cells is shown in (a-c). The bars represent the mean ± standard deviation of two independent experiments; *P < 0.05, **P < 0.005, and ***P < 0.0005 in comparison with untreated cells. Type I collagen present in culture supernatants was quantified by immunoblotting (d) and TIMP-1 by enzyme-linked immunosorbent assay (e). Bars represent the increase in protein levels in treated cells relative to untreated cells. A representative identification of type I collagen protein by Western blotting is inserted in (d). Bort, bortezomib; *COL1A*, collagen 1A1; TGF-β, transforming growth factor-beta; TIMP-1, tissue inhibitor of matrix metalloproteinase-1; UT, untreated.

We previously reported that PI decreases type I collagen and TIMP-1 production in human dermal fibroblasts [21]. Since type I collagen has a trimeric structure composed of two alpha1 subunits and one alpha2 subunit, we explored whether PI affected the transcription of both the *COL1A1 *and *COL1A2 *genes. Bortezomib decreased both *COL1A1 *(10-fold) and *COL1A2 *(5-fold) steady-state mRNA levels in a time-dependent manner (Figure 1a, b), as assessed by quantitative PCR. Bortezomib had only modest effects on *TIMP-1 *mRNA levels (Figure 1c). As expected, TGF-β induced a time-dependent increase in *COL1A1 *and *COL1A2 *mRNA levels (Figure 1a-c). Interestingly, the addition of bortezomib 1 hour before TGF-β completely abolished the effect of TGF-β on *COL1A1 *and *COL1A2 *(Figure 1a, b). Bortezomib also inhibited, albeit to a lesser extent, TGF-β-induced *TIMP-1 *transcription (Figure 1c). Consistent with these results, bortezomib strongly inhibited the production of type I collagen and TIMP-1 proteins induced by TGF-β (Figure 1d, e).

### TGF-β activates the transcription of *COL1A1 *and *COL1A2 *via AP2 and SP1 binding sites, respectively

**Figure 2:**
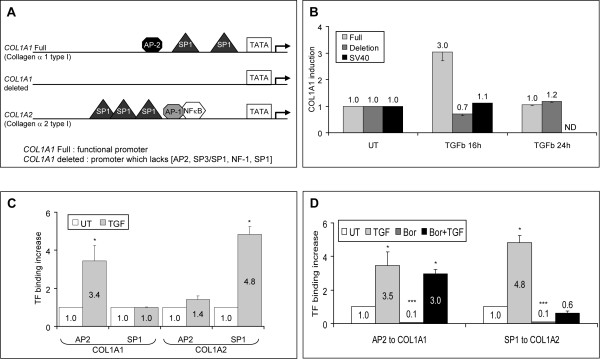
**Proteasome inhibition abolishes the increased binding of SP1 to *COL1A2 *but not of AP2 to *COL1A1 *promoter induced by TGF-β**. **(a) **Schematic representation of the *COL1A1 *and *COL1A2 *promoter regions and the deleted *COL1A1 *construct. **(b) **Dermal fibroblasts were transiently transfected with luciferase reporter gene constructs carrying the SV40 promoter, full-length *COL1A1 *promoter, deleted *COL1A1 *promoter, or no promoter. Luciferase activity was measured after TGF-β treatment (16 or 24 hours) and normalized to the levels obtained with cells transfected with the promoter-free construct. Histograms show the increase in *COL1A1 *promoter activity in treated cells relative to untreated cells. The results represent the mean ± standard deviation (SD) of two independent experiments. **(c, d) **Fibroblasts were treated with TGF-β (5 ng/mL) for 4 hours or bortezomib (1 μM) for 16 hours or both (TGF-β was added 1 hour after bortezomib) for 4 hours or were left untreated. Crosslinked chromatin was extracted, sonicated, and immunoprecipitated with anti-AP2 or anti-SP1 antibodies. Transcription factor-bound DNA fragments were quantified by real-time polymerase chain reaction using the primers indicated in Table 1. The increase in treated cells relative to untreated cells is shown. The bars represent the mean ± SD of two independent experiments; *P < 0.05 and ***P < 0.0005 in comparison with untreated cells. Bor, bortezomib; *COL1A*, collagen 1A; ND, not determined; TF, transcription factor; TGF-β, transforming growth factor-beta; UT, untreated.

Transcriptional activation of the *COL1A2 *gene by TGF-β is controlled mainly by SP1 binding sites in its promoter region [9] (Figure 2a), whereas TGF-β activation of the *COL1A1 *gene in human dermal fibroblasts remains poorly understood. To partially characterize the requirements for TGF-β responsiveness, we performed luciferase reporter gene assays in human dermal fibroblasts to compare the activity of a full-length *COL1A1 *promoter with that of a *COL1A1 *promoter lacking AP2, SP3/SP1, NF-1, and SP1 binding sites [32] (Figure 2a). TGF-β stimulated the full-length *COL1A1 *promoter, resulting in a threefold increase in the transcriptional activity after 16 hours of incubation. This increase was transient and lost by 24 hours of incubation (Figure 2b). Interestingly, TGF-β had no effect on the activity of the deleted *COL1A1 *promoter or the constitutive SV40 promoter used as negative control. These experiments indicate that at least one of the transcription factor binding sites (AP2, SP3/SP1, NF-1, SP1) present in the promoter proximal region is required for TGF-β induction of *COL1A1 *transcription. To assess whether the observed increase in *COL1A1 *promoter activity induced by TGF-β correlated with an *in vivo *increase in the binding of specific transcription factors, we performed chromatin immunoprecipitation (ChIP) experiments using SP1- and AP2-specific anti-sera. TGF-β induced a substantial increase in binding of AP2, but not of SP1, to the *COL1A1 *promoter (Figure 2c). This was distinctly different from the observed increase in binding of SP1, but not of AP2, to the promoter region of *COL1A2 *in response to TGF-β (Figure 2c). Thus, the coordinated increase in *COL1A1 *and *COL1A2 *gene transcription in response to TGF-β is mediated, at least in part, by distinct transcription factors.

### Proteasome inhibition abolishes TGF-β-induced SP1 binding to *COL1A2 *but not of AP2 to *COL1A1*

We next determined whether PI could affect binding of the identified transcription factors involved in type I collagen gene activation by TGF-β. We quantified binding of AP2 and SP1 to the *COL1A1 *and *COL1A2 *promoter regions in fibroblasts cultured in the presence of TGF-β or bortezomib or both. As expected, TGF-β increased binding of AP2 to COL1A1 and SP1 to COL1A2. Conversely, bortezomib potently inhibited the binding of AP2 and SP1 to their respective promoter regions in unstimulated fibroblasts (Figure 2d). Of major interest, bortezomib abolished TGF-β-induced binding of SP1 to COL1A2 (0.6- versus 4.8-fold) but failed to significantly affect TGF-β-induced binding of AP2 to COL1A1 (3.0- versus 3.5-fold) (Figure 2d). Thus, *COL1A1 *and *COL1A2 *promoter regions are bound by different transcription factors in response to TGF-β and their binding is affected differently by PI.

### Proteasome inhibition does not affect TGF-β-induced Smad2 phosphorylation, nuclear translocation, or binding to the *COL1A2 *promoter

**Figure 3:**
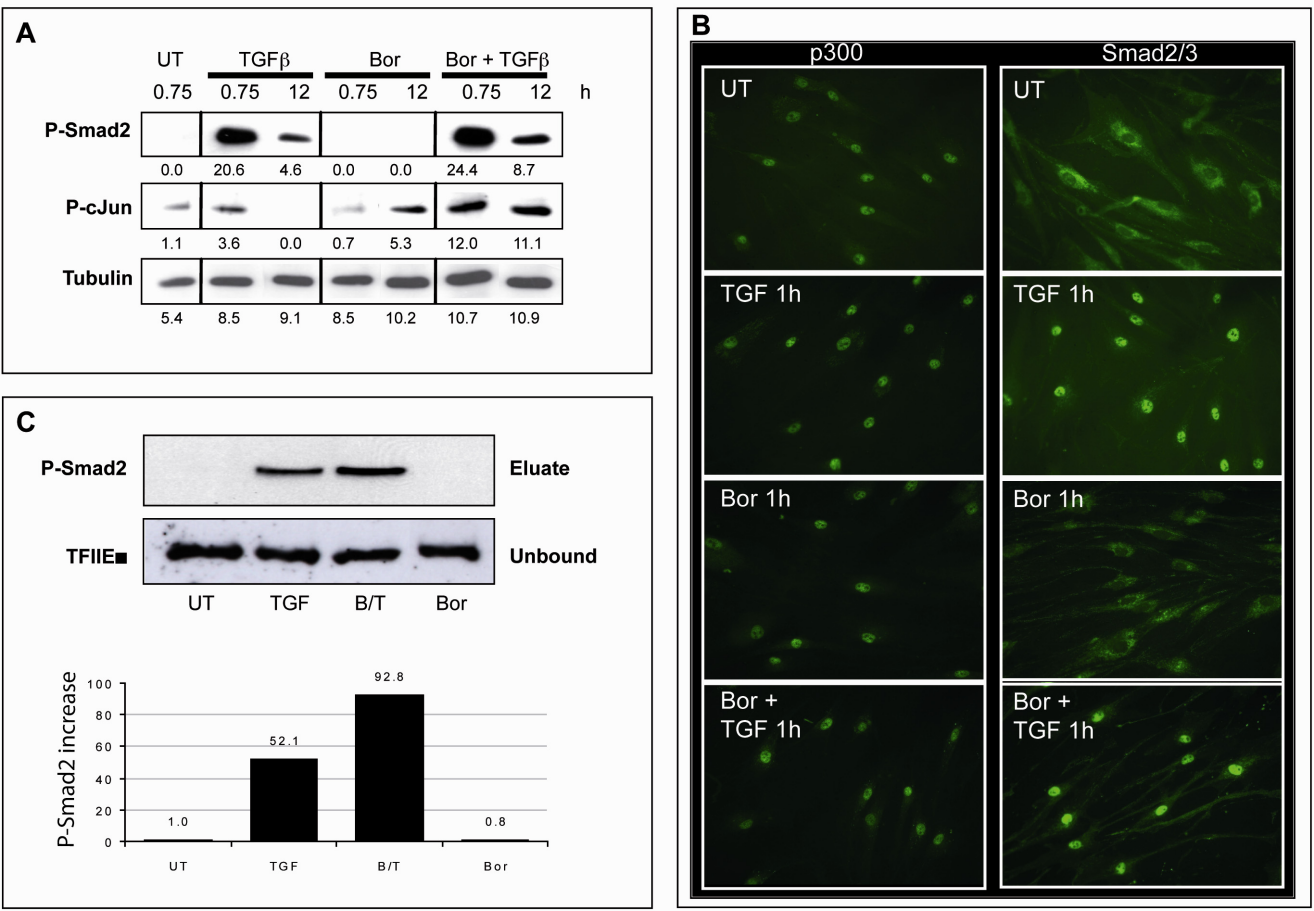
**Bortezomib does not abrogate TGF-β-induced phosphorylation, nuclear translocation, or binding of Smad2 to the *COL1A2 *promoter**. Fibroblasts were treated with TGF-β (5 ng/mL) or bortezomib (1 μM) or both (TGF-β was added 1 hour after bortezomib) or were left untreated for the indicated amount of time. **(a) **Total protein extracts were analyzed by Western blotting. Band intensities are provided below. **(b) **Fibroblasts were labeled with rabbit anti-p300 or Smad2/3 antibodies. Immunofluorescence photographs (× 40) from one representative experiment out of three independent experiments are presented. **(c) **Nuclear proteins from fibroblasts were extracted and used to perform DNA pull-down assays. DNA-bound proteins were eluted and analyzed by Western blotting using anti-P-Smad2 antibodies. Total nuclear protein content was assessed using anti-TFIIEα antibodies on unbound fractions. Band intensities were quantified and normalized to those obtained with the anti-TFIIEα antibody. The increase in P-Smad2 levels in treated relative to untreated cells is provided. Bor, bortezomib; B/T, bortezomib/transforming growth factor-beta; *COL1A2*, collagen 1A2; TGF-β, transforming growth factor-beta; UT, untreated.

TGF-β-mediated activation of the *COL1A2 *gene is triggered by binding of a transcription factor complex comprising SP1, p300, and Smad2/3 to the SP1 sites in its promoter region [9]. We were interested in investigating whether the inhibitory effect of PI on SP1 binding to *COL1A1 *could be due to its effects on Smad2 signaling. We therefore studied the fate of Smad2 in fibroblasts cultured in the presence of TGF-β or bortezomib or both. As expected, TGF-β triggered a pronounced phosphorylation of Smad2 in dermal fibroblasts. This phosphorylation was strong at 45 minutes and then decreased, although it remained detectable after 12 hours (Figure 3a). Bortezomib did not induce Smad2 phosphorylation on its own and did not modify Smad2 phosphorylation induced by TGF-β (Figure 3a). Bortezomib was active, however, since c-Jun phosphorylation was induced as expected [21] at late time points (Figure 3a).

Upon phosphorylation, Smad2 is known to translocate into the nucleus in response to TGF-β [34]. Pre-incubation of fibroblasts with bortezomib did not alter the cytoplasmic pattern of Smad2 in resting fibroblasts or its nearly complete nuclear translocation at 1 hour after the addition of TGF-β (Figure 3b). Furthermore, bortezomib did not alter the exclusive nuclear localization of p300, regardless of the presence or absence of TGF-β (Figure 3b). Finally, using a synthetic biotinylated probe (sequence in Table 1) in a pull-down assay, we quantified binding of phospho-Smad2 to the SP1 sequence of the *COL1A2 *promoter. In these experiments, TGF-β induced a substantial increase in binding of phospho-Smad2. The simultaneous presence of bortezomib did not reduce but instead enhanced binding of phospho-Smad2 in response to TGF-β, while bortezomib on its own did not induce any binding (Figure 3c). Taken together, these findings demonstrate that PI does not alter TGF-β-induced Smad2 phosphorylation, nuclear translocation, or binding to the *COL1A2 *promoter. Impaired Smad2 activation thus cannot explain the reduced production of collagen when fibroblasts are stimulated by TGF-β in the presence of PI.

### Differential effects of proteasome inhibition and TGF-β on MMP-1 and MMP-2

**Figure 4:**
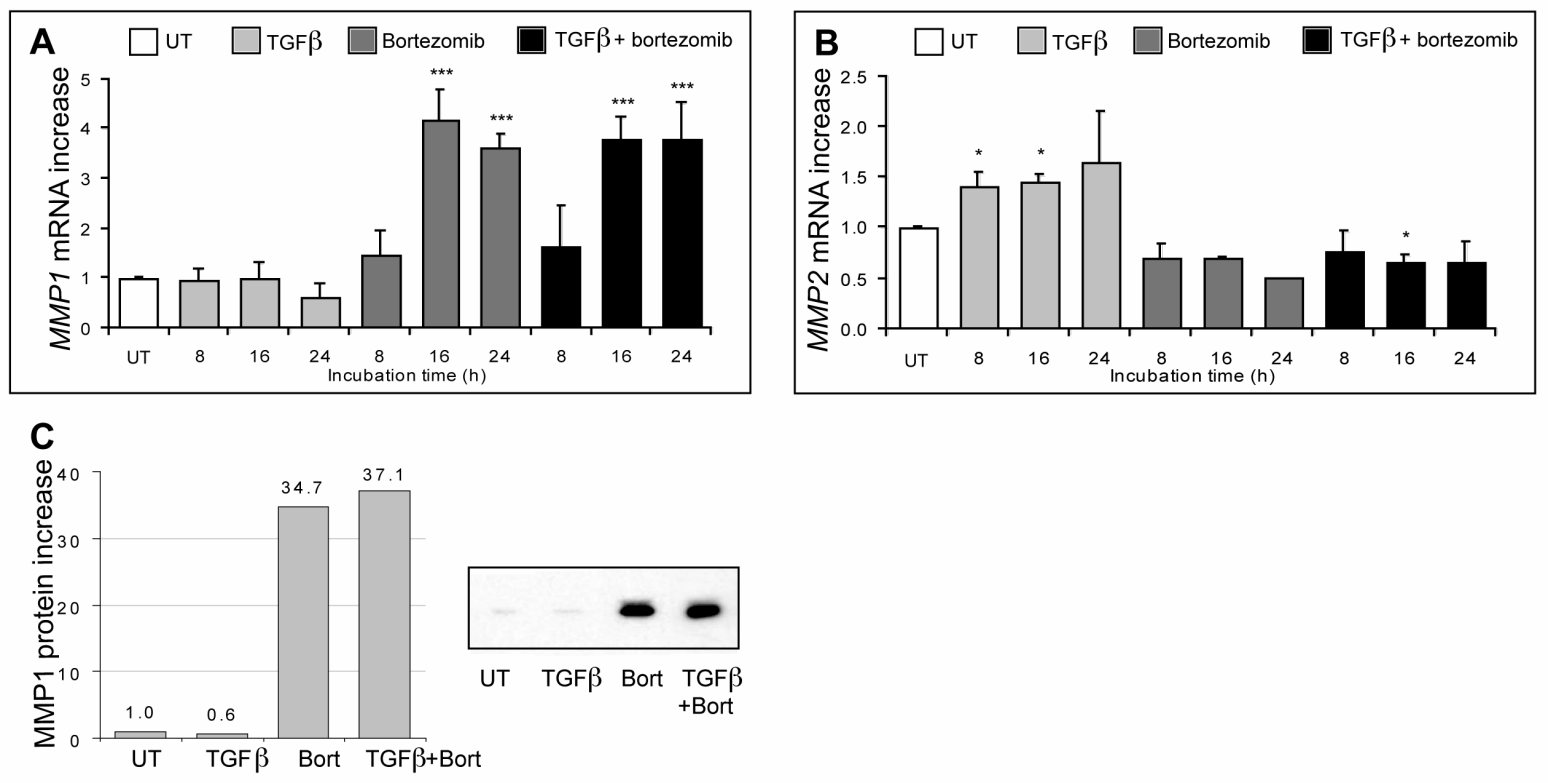
**Variations in MMP-1 and MMP-2 expression upon treatment of dermal fibroblasts with bortezomib or TGF-β or both**. mRNA and protein extracts from treated fibroblasts were processed as described in the legend of Figure 1. The increases in mRNA levels for *MMP-1 ***(a) **and *MMP-2 ***(b) **are reported. The data represent the mean ± standard deviation of two independent experiments; *P < 0.05 and ***P < 0.0005 in comparison with untreated cells. **(c) **Protein levels were quantified by immunoblotting for MMP-1. The increase in protein levels in the treated cells relative to untreated cells is shown. The analysis of MMP-1 protein by Western blotting is inserted in the upper panel. Data are from a representative experiment. Bort, bortezomib; MMP, matrix metalloproteinase; TGF-β, transforming growth factor-beta; UT, untreated.

MMPs play a major role in ECM degradation and are differentially regulated by TGF-β, which was reported to decrease MMP-1 and increase MMP-2 production by fibroblasts [16,18]. We were therefore interested in investigating the effect of PI on MMP production in the presence or absence of TGF-β. We confirmed that bortezomib stimulated MMP-1 production and that this increase was dominant over the inhibitory effect of TGF-β, both at the mRNA and protein levels (Figure 4a, c) [21]. Furthermore, bortezomib modestly decreased basal and TGF-β induced MMP-2 mRNA expression (Figure 4b). Thus, PI differentially affects the regulation of MMP-1 and MMP-2 production in fibroblasts. It should be emphasized that the MMP-1 mRNA half-life in the presence of the transcriptional inhibitor 5,6-dichlorobenzimidazole riboside (DRB) with and without proteasome inhibitor exceeded 24 hours (data not shown). However, steady-state MMP-1 mRNA levels increased in the presence of PI (Figure 4a). It is thus highly unlikely that MMP-1 mRNA stability is significantly affected by PI.

### Proteasome inhibition activates the *MMP*-1 promoter by inducing binding of c-Jun to the proximal AP-1 site

**Figure 5:**
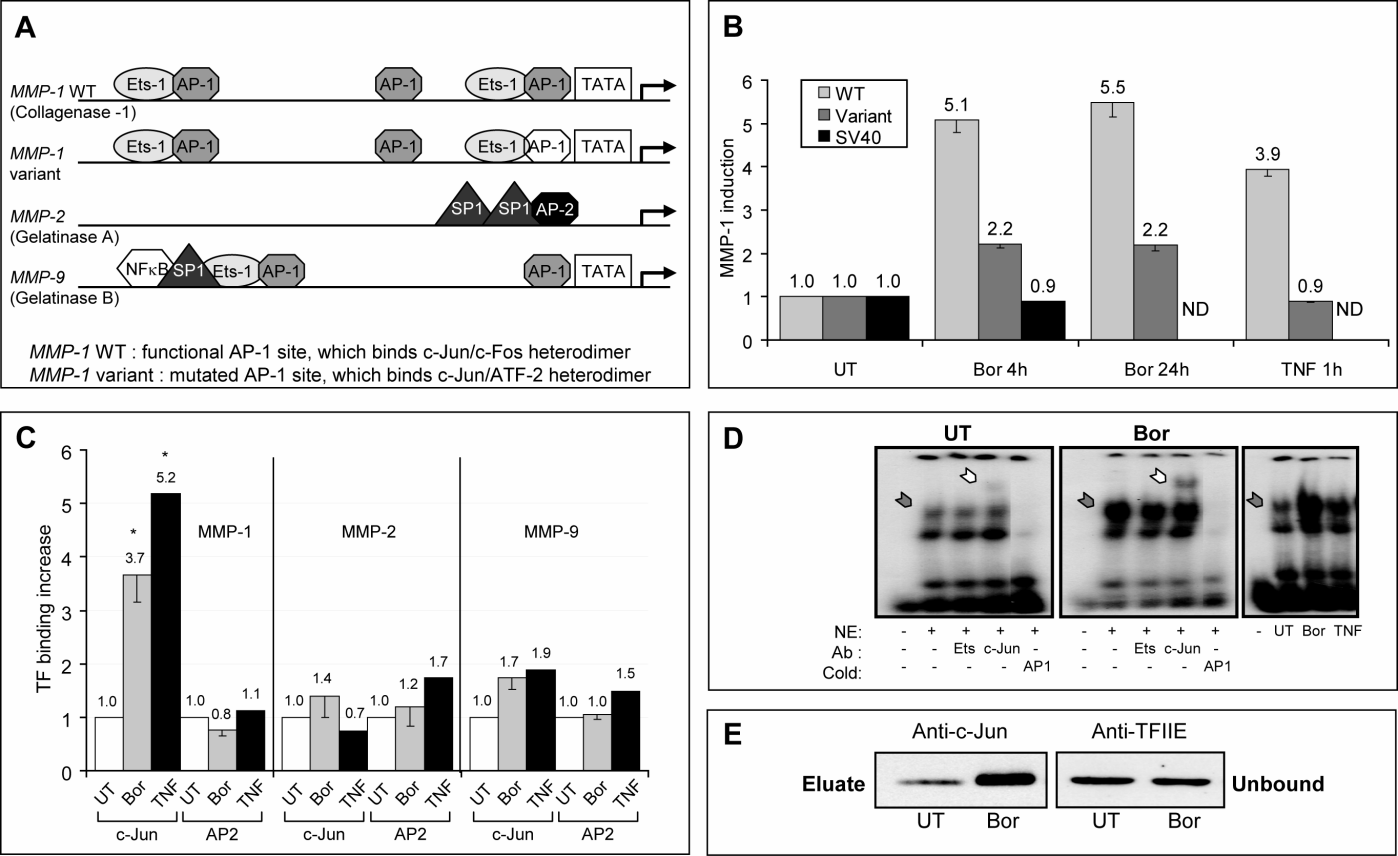
**Bortezomib activates the *MMP-1 *promoter via binding of c-Jun**. **(a) **Schematic representation of *MMP *promoter regions. Transcription factor (TF) binding sites and TATA boxes are shown (adapted from [35]). **(b) **Luciferase reporter gene experiments were performed as described in Figure 2b, except that the constructs carried the SV40, wild-type, or variant *MMP-1 *promoter or no promoter. Luciferase activity was measured after bortezomib (4 or 24 hours) or TNF-α (1 hour) treatment, normalized, and reported as in Figure 2b. The results represent the mean ± standard deviation (SD) of two independent transfections. **(c) **Fibroblasts were treated for 1 hour with TNF-α (10 ng/mL) or for 16 hours with bortezomib (1 μM). Chromatin immunoprecipitation was performed with anti-c-Jun or anti-AP2 antibodies, and the results were quantified by real-time polymerase chain reaction using the primers indicated in Table 1. The increase in binding of c-Jun or AP2 to the *MMP *promoters in treated cells relative to untreated cells is shown. The results represent the mean ± SD of three independent experiments; *P < 0.05 in comparison with untreated cells. **(d, e) **Nuclear extracts were prepared from fibroblasts that were treated for 16 hours with 1 μM bortezomib or 1 hour with 10 ng/mL TNF-α or that were left untreated. Binding of TF to a synthetic AP-1 site was assessed by electrophoretic mobility shift assay (d) using a specific radiolabeled probe in the presence (+) or absence (-) of anti-Ets or anti-c-Jun antibodies or cold probe (AP-1). A gray arrow indicates band shift, and a white arrow indicates supershifted band. Alternatively, TF-DNA binding was assessed by a DNA pull-down assay (e) using a biotinylated MMP-1 probe and anti-c-Jun antibodies. Total nuclear protein content was assessed using anti-TFIIEα antibodies on unbound fractions. Bor, bortezomib; MMP, matrix metalloproteinase; ND, not determined; NE, nuclear extract; TNF-α, tumor necrosis factor-alpha; UT, untreated; WT, wild-type.

To identify the promoter sequences that are necessary and sufficient for driving the induction of *MMP-1 *in response to PI, we assessed the impact of bortezomib on fibroblasts transiently transfected with reporter gene constructs carrying either an intact *MMP-1 *promoter or a mutated MMP-1 promoter in which a binding site for c-Jun/c-Fos was replaced by a c-Jun/ATF-2 binding site (Figure 5a and Table 1) [31]. In the presence of bortezomib, a 5.1-fold increase in activity of the intact *MMP-1 *promoter was observed at 4 hours (Figure 5b). This increase was similar in magnitude to that observed in cells treated with TNF-α (3.9-fold), which was used as a positive control. Interestingly, the increase in MMP-1 promoter activity was long-lasting and remained high after 24 hours of culture (Figure 5b). Mutation of the AP-1 site in the promoter resulted in a substantial reduction in *MMP-1 *induction by bortezomib (2.2-fold at 4 and 24 hours) and unresponsiveness to TNF-α (0.9-fold) (Figure 5b). This demonstrates that optimal bortezomib-induced activation of *MMP-1 *transcription requires an AP-1 binding site recognized preferentially by a c-Jun/c-Fos heterodimer.

To confirm the involvement of c-Jun in bortezomib-mediated induction of *MMP-1 *transcription, we assessed its *in vivo *binding to the promoter region of the *MMP-1 *gene by ChIP. We focused on the promoter proximal region on the basis of published regulatory sites in the *MMP-1 *promoter (Figure 5a) [35] and the results we obtained using the mutated *MMP-1 *promoter. Bortezomib provoked a strong increase in binding of c-Jun to the promoter region, comparable to that induced by TNF-α (3.7- versus 5.2-fold) (Figure 5c). Enhanced binding of c-Jun to the *MMP-1 *promoter was specific as the AP2 transcription factor did not exhibit an increase in binding upon either treatment, and binding of c-Jun to the *MMP-2 *or *MMP-9 *promoters was not enhanced (Figure 5c). Thus, the differential effect of bortezomib on MMP-1 and MMP-2 expression is dependent, at least in part, on the specific characteristics of their promoter regions.

**Figure 6:**
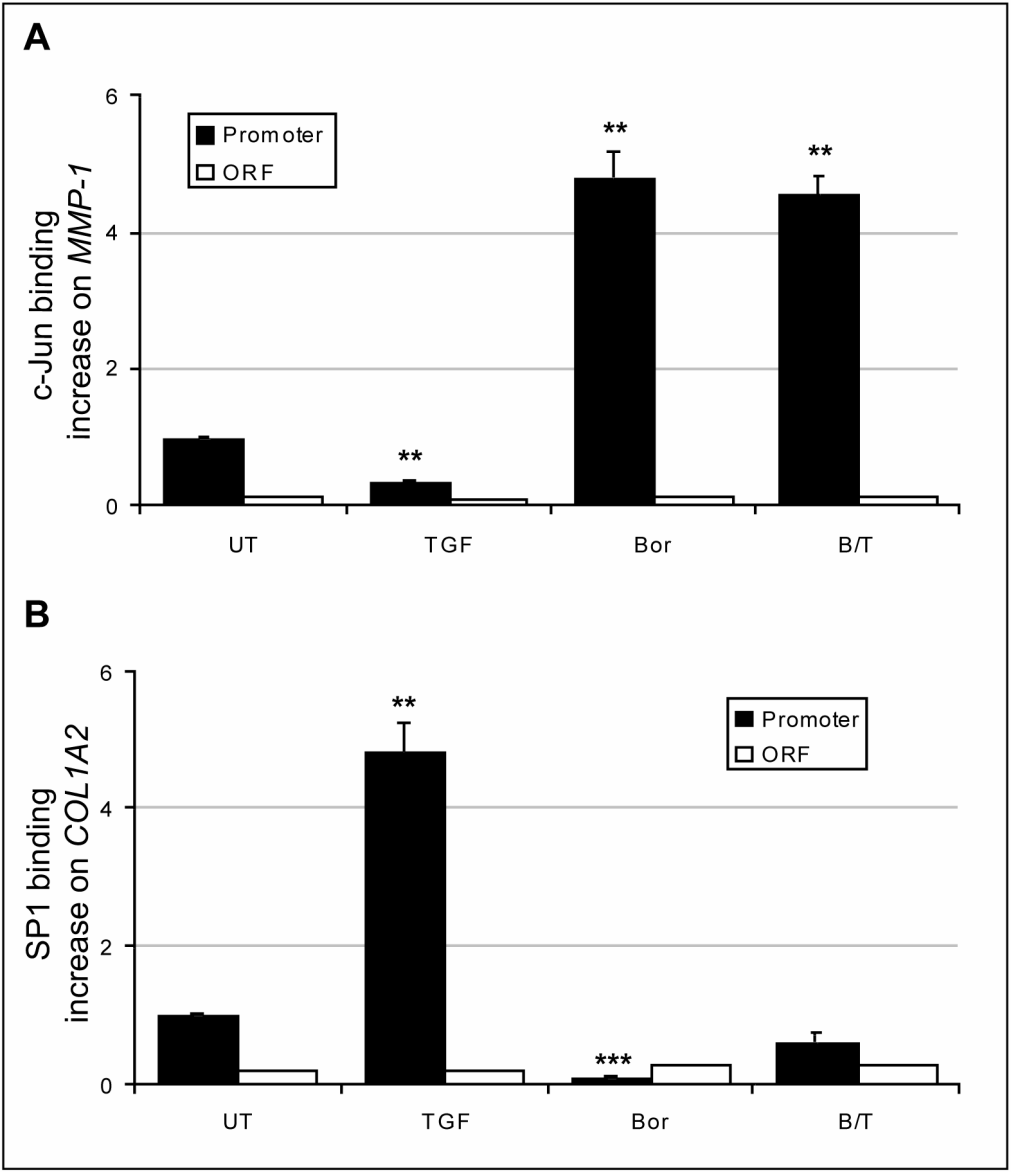
**Bortezomib and TGF-β exert opposing regulation on *COL1A *and *MMP-1 *genes**. Fibroblasts were treated with TGF-β (5 ng/mL) for 4 hours or bortezomib (1 μM) for 16 hours or both (TGF-β was added 1 hour after bortezomib) for 4 hours or were left untreated. Chromatin immunoprecipitations were performed with anti-c-Jun and anti-SP1 antibodies, and the results were quantified by real-time polymerase chain reaction using primers hybridizing the promoter (black bar) or the open reading frame (ORF) (white bar) region of either gene (Table 1). The increases in binding of c-Jun to the *MMP-1 *promoter **(a) **and of SP1 to the *COL1A2 *promoter **(b) **in treated cells relative to untreated cells are reported. Specificity of the experiments was controlled by assessing binding of c-Jun or SP1 to the ORF of the *MMP-1 *(a) and *COL1A2 *(b) genes, respectively. The data represent the mean ± standard deviation of three independent experiments; **P < 0.005 and ***P < 0.0005 in comparison with untreated cells. Bor, bortezomib; B/T, bortezomib/transforming growth factor-beta; *COL1A*, collagen 1A; MMP, matrix metalloproteinase; TGF-β, transforming growth factor-beta; UT, untreated.

The *MMP-1 *promoter contains at least two proximal AP-1 binding sites, separated by less than 100 nucleotides. Based on the literature, we postulated that the most proximal site was likely to be the bortezomib-responsive element. We therefore performed electrophoretic mobility shift assays (EMSAs) with double-stranded oligonucleotides corresponding to the most proximal AP-1 site of the *MMP-1 *promoter (Table 1 for oligonucleotide sequences). Incubation of the MMP-1 probe with nuclear extracts from untreated fibroblasts (Figure 5d, left panel) led to the formation of two protein-DNA complexes, of which only the upper band was modulated by treatment (gray arrow), demonstrating the binding of one or several transcription factors to this synthetic DNA sequence. Competition with cold oligonucleotides demonstrated the specificity of this binding. The presence of c-Jun among the bound proteins was indicated by the appearance of a supershifted band (white arrow) upon pre-incubation with anti-c-Jun antibodies. No supershift was observed in the presence of control anti-Ets-1 antibodies (Figure 5d, middle panel). Similar patterns were obtained with nuclear extracts generated from bortezomib- and TNF-α-treated fibroblasts (Figure 5d, right panel). Finally, DNA pull-down experiments revealed a marked increase in the amount of c-Jun bound to the biotinylated MMP-1 probe upon bortezomib treatment (Figure 5e). Taken together, these experiments demonstrate that PI results in a specific increase in binding of c-Jun to the most proximal AP-1 site of the *MMP-1 *promoter. We next investigated whether binding of c-Jun to the *MMP-1 *promoter in the presence of bortezomib was regulated by TGF-β. This was not the case since ChIP experiments revealed that bortezomib-induced binding of c-Jun to the *MMP-1 *promoter was not affected by TGF-β (Figure 6a). Of note, under the same culture conditions, TGF-β-induced binding of SP1 to the promoter region of *COL1A2 *was abrogated by bortezomib (Figure 6b). In control experiments, binding of c-Jun did not increase at the *COL1A2 *promoter, nor did binding of SP1 at the *MMP-1 *promoter (data not shown). Furthermore, the specificity of our ChIP assays was emphasized by the fact that no binding of c-Jun or SP1 was observed within the open reading frames (ORFs) of the *MMP-1 *or *COL1A2 *genes (Figure 6a, b). In conclusion, the effect of PI dominates the influence of TGF-β in controlling the binding of both SP1 to *COL1A2 *and c-Jun to *MMP-1*.

## Discussion

**Figure 7:**
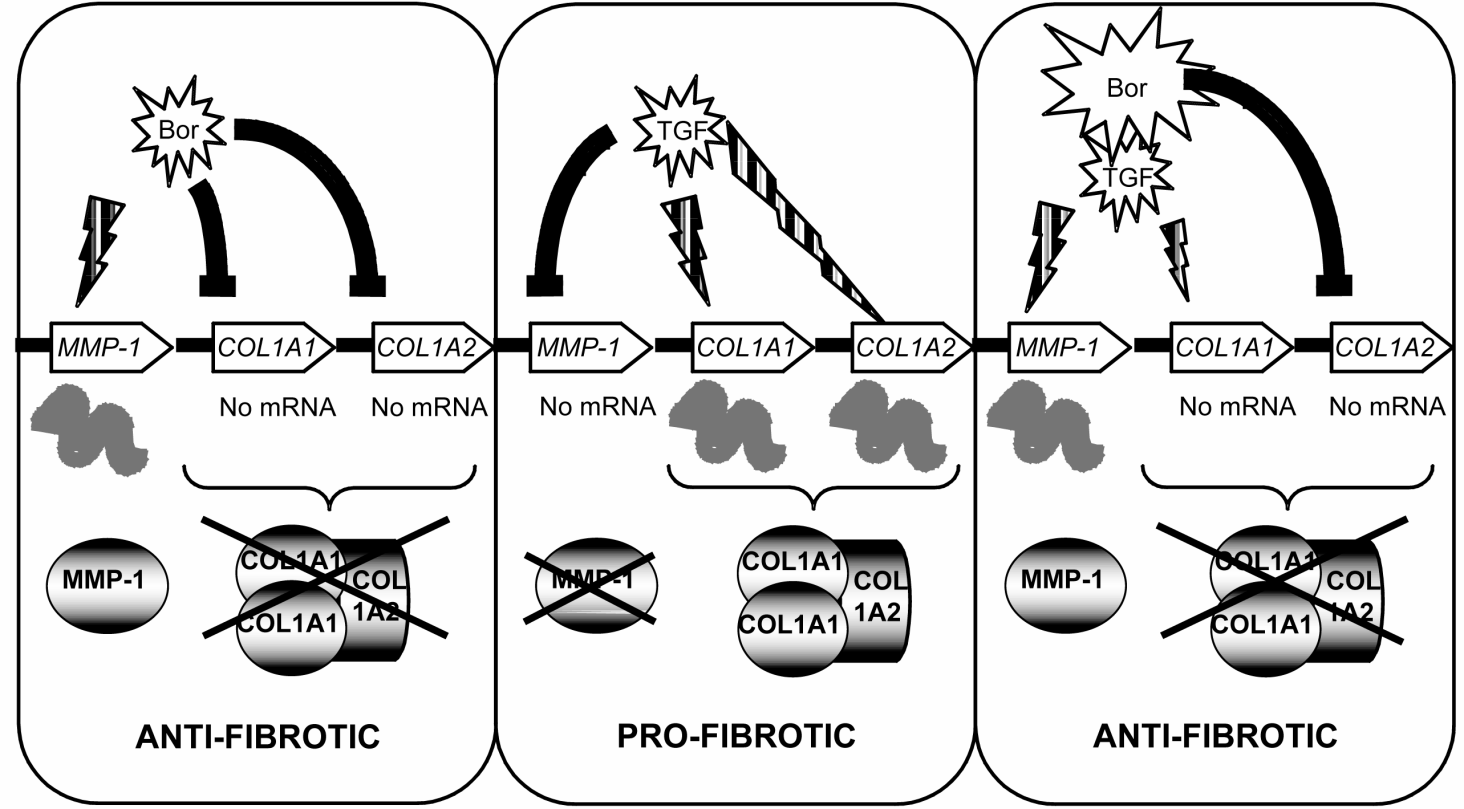
**Bortezomib overrides the effect of TGF-β and imposes its anti-fibrotic activity**. Schematic representation of the effect of bortezomib (left), TGF-β (middle), and bortezomib plus TGF-β (right) on transcription of the *MMP-1*, *COL1A1*, and *COL1A2 *genes. See the Discussion section of the text for details. Bor, bortezomib; *COL1A*, collagen 1A; MMP-1, matrix metalloproteinase-1; TGF-β, transforming growth factor-beta.

The major finding of our work is that the overall anti-fibrotic activity of PI by bortezomib results from two distinct but functionally converging regulatory effects summarized in Figure 7. On one hand, we have documented that enhanced transcription of the *MMP-1 *gene depends on enhanced binding of c-Jun to the most proximal AP-1 binding site of the *MMP-1 *promoter. On the other hand, we have shown that inhibition of SP1 binding to the promoter of *COL1A2 *correlates with decreased *COL1A2 *transcription in both unstimulated and TGF-β-stimulated fibroblasts. It is noteworthy that these promoter elements were previously shown to be important for regulating the transcription of *MMP-1 *[35] and *COL1A2 *[9,36], respectively.

The stimulatory effect we observed on MMP-1 expression was specific to this particular MMP since bortezomib decreased MMP-2 transcription. This could be explained by the absence of AP-1 binding sites in the MMP-2 promoter (Figure 5a). However, the *MMP-1 *promoter shares highly conserved consensus AP-1 binding sequences with other *MMP *promoters, including MMP-9 (Figure 5a), which may suggest that the activation by bortezomib is not restricted to MMP-1. While we have not directly assessed whether MMP-9 transcription was enhanced by PI, we did not observe enhanced binding of c-Jun to the AP-1 site in the promoter of MMP-9. This may be due to the influence of sequences adjacent to the AP-1 site or to the composition of the AP-1 complex that binds there, and this complex could correspond to c-Jun homodimers or heterodimers of c-Jun with JunB, JunD, or c-Fos. In particular, a notable difference is the presence of an Ets-1 site next to the distal AP-1 site (-1604) in *MMP-1 *but not in *MMP-9*. In agreement with previous observations [37], we postulate that the presence of an Ets-1 site next to an AP-1 sequence enhances the binding of c-Jun to the AP-1 site, thereby increasing transcription of the downstream gene. This is consistent with the finding that bortezomib-induced MMP-1 transcription is reduced in reporter gene assays when the Ets-1 site in the MMP-1 promoter is mutated.

Our investigation of the effect of bortezomib on type I collagen synthesis revealed that reduced *COL1A2 *transcription correlated with decreased binding of SP1 to the *COL1A2 *promoter. This was observed under both basal and TGF-β-induced conditions. Since we previously demonstrated that PI did not affect COL1A1 mRNA stability [21], decreased transcription explains the PI effect. SP1 is known to be a crucial cis-acting element for basal COL1A2 transcription and also to play an important role in mediating TGF-β-induced transcription [8,38]. Furthermore, hyper-phosphorylation of SP1 is characteristic of SSc dermal fibroblasts [39]. It is therefore likely that the effect of bortezomib on type I collagen synthesis is mediated, at least in part, by its capacity to reduce SP1 binding to the promoter of COL1A2. We were unable, however, to link decreased SP1 binding to upstream events. In particular, we tested the hypothesis that reduced SP1 binding might be associated with an effect of bortezomib on canonical Smad signaling in response to TGF-β. Indeed, recent studies on TGF-β signaling have revealed the ability of Smads to interact with various components of the 26S proteasome system [40]. Such interactions are now known to contribute to the regulation of Smad protein levels before and after Smad activation [41]. Most importantly, such interactions have also been shown to contribute to the signaling functions of Smads. This involves interactions with several proteins, such as Smad ubiquitination regulatory factors (Smurfs), the oncoprotein SnoN, and the multi-domain docking protein HEF1. Proteasomal degradation of these proteins links TGF-β signaling to multiple signaling pathways [42]. In our experimental conditions, however, bortezomib did not affect Smad2 phosphorylation or nuclear translocation and actually increased its binding to the COL1A2 promoter. In this context, it could be speculated that increased affinity of a single factor can have a negative effect on transcription, and this may explain the negative effect of bortezomib on COL1A2 synthesis. TGF-β-stimulated transcription of *COL1A2 *is triggered by binding of a large transcription factor complex, which is composed of Smad2/3, Smad4, SP1, and the transcription factor p300 [43]. In this complex, Smad2/3 has been reported to interact directly with both SP1 and p300 [44]. Although no direct interaction between SP1 and p300 has been reported at the *COL1A2 *promoter, a recent study demonstrated that SP1 binds p300 and recruits it for *NECL1 *transcription [45]. Since bortezomib did not prevent Smad2 binding to *COL1A2*, it can be hypothesized that it affects SP1 directly or perturbs in a more subtle manner the interactions of SP1 with Smad2/3 or p300. In this respect, it is interesting to note that p300/CBP sequestration by c-Jun or STAT1 has been proposed to explain, at least in part, the antagonism exerted on collagen synthesis by TNF-α and interferon-gamma, respectively [44,46,47]. Thus, c-Jun, which we have demonstrated to be increased in PI-treated fibroblasts [21], is known to participate in the functional availability of p300 [48,49]. In addition, PI has been shown to affect the histone acethyltransferase activity of p300 [50,51], which could affect binding of transcription factors to the *COL1A2 *promoter. Finally, off-DNA complexes formed by the increased availability of c-Jun with other specific or general transcription factors may be at play. In the present work, we have not directly assessed the effect of bortezomib on SSc fibroblasts. However, in terms of type I collagen and MMP-1 protein production, SSc and control fibroblasts were previously found to behave similarly when submitted to PI [21].

## Conclusions

By altering the binding of at least two transcription factors, c-Jun and SP1, PI results in increased production of MMP-1 and decreased synthesis of type I collagen in human dermal fibroblasts, thus providing a novel rationale for assessing the potential of drugs targeting the proteasome for their anti-fibrotic properties.

## Abbreviations

AP-1: activation protein-1; CBP: CREB-binding protein; ChIP: chromatin immunoprecipitation; *COL1A*: collagen 1A; DTT: dithiothreitol; ECM: extracellular matrix; EDTA: ethylenediaminetetraacetic acid; EMSA: electrophoretic mobility shift assay; FCS: fetal calf serum; MMP: matrix metalloproteinase; PBS: phosphate-buffered saline; PCR: polymerase chain reaction; PI: proteasome inhibition; PMSF: phenylmethylsulfonyl fluoride; SSc: systemic sclerosis; TGF-β: transforming growth factor-beta; TIMP: tissue inhibitor of matrix metalloproteinase; TNF-α: tumor necrosis factor-alpha.

## Competing interests

The authors declare that they have no competing interests.

## Authors' contributions

LG conceived experiments, performed research, analyzed the data, and drafted the manuscript. QS-E conceived experiments and performed research. MA performed research. WR conceived experiments and critically revised the manuscript. CC conceived research, analyzed the data, and drafted the manuscript. All authors read and approved the final manuscript.
